# Physical Behavior Profiles in Chronic Cancer-Related Fatigue

**DOI:** 10.1007/s12529-017-9670-3

**Published:** 2017-07-11

**Authors:** M. D. J. Wolvers, J. B. J. Bussmann, F. Z. Bruggeman-Everts, S. T. Boerema, R. van de Schoot, M. M. R. Vollenbroek-Hutten

**Affiliations:** 1 Telemedicine Group, Roessingh Research and Development, Enschede, the Netherlands; 2 Telemedicine Group, Faculty of Electrical Engineering, Mathematics and Computer Science, University of Twente, Enschede, the Netherlands; 3 Department of Rehabilitation Medicine, Erasmus MC University Medical Center, Rotterdam, the Netherlands; 4 Scientific Research Department, Helen Dowling Institute, Bilthoven, the Netherlands; 5 Department of Methods and Statistics, Utrecht University, Utrecht, the Netherlands; 6 Optentia Research Program, Faculty of Humanities, North-West University, Vanderbijlpark, South Africa

**Keywords:** Cancer, Oncology, Latent profiles analysis, Fatigue, Accelerometry, Physical behavior

## Abstract

**Purpose:**

Increasing physical activity level is a generally effective intervention goal for patients who suffer from chronic cancer-related fatigue (CCRF). However, patients are unlikely to benefit equally from these interventions, as their behavioral starting points might vary substantially. Therefore, we explored patterns of physical behavior of participants who suffer from CCRF.

**Methods:**

Baseline data of a randomized controlled trial were used for a latent profile analysis on nine accelerometer-derived physical behavior measures, describing levels and patterns of physical activity, moderate-to-vigorous intensity physical activity (MVPA), and sedentary behavior. The relation between participant characteristics and the latent profiles was analyzed.

**Results:**

Accelerometer data of 172 participants from the Netherlands was analyzed. Three latent profiles were distinguished that differed most on physical activity level and total time spent in MVPA. Eighty-eight percent of all participants were assigned to a profile with a probability higher than 8. Age and perceiving limitations by comorbid conditions and pain were significant covariates of profile membership.

**Conclusions:**

We distinguished three physical behavior profiles. The differences between the patterns indicate that the heterogeneity of this sample requires patients to have substantially different treatment goals. Further research should test the applicability of these profiles in clinical practice.

**Electronic supplementary material:**

The online version of this article (doi:10.1007/s12529-017-9670-3) contains supplementary material, which is available to authorized users.

## Introduction

Fatigue is a common and debilitating side effect of cancer and its treatment that often persists well beyond active cancer treatment [1, 2]. Chronic cancer-related fatigue (CCRF) prevents patients to have “a normal life” [3] and hampers in performing daily activities [4].

The role of physical activity in the context of chronic cancer-related fatigue is neither straightforward nor evident. For example, inactivity has been proposed as a result as well as a cause of fatigue. Lower physical activity was associated with fatigue before initiation of treatment [5] and with persistence of fatigue [1]. However, a large cohort study of Neil et al. [6] showed no difference in inactivity between cancer survivors and individuals with no history of cancer, and another study showed that self-reported physical activity was no predictor for fatigue before and after chemotherapy [7].

Behavioral interventions that try to reduce fatigue usually aim at increasing level of physical activity by means of exercise or graded activity [8–11]. These interventions are generally effective in reducing fatigue [8–12]. Such interventions should be adapted to individual physiological differences [13] to be fully appreciated. Presumably, effective interventions correspondingly depend on the individual’s starting point in terms of physical behavior. Therefore, it is needed to consider the heterogeneity of patients’ physical behavior and to explore what patient characteristics relate to these behaviors.

In fact, heterogeneity in physical behavior has been scarcely considered in behavioral intervention studies. One example is a study by Van der Werf et al. who aimed to reveal heterogeneity of physical behavior in non-cancer patients who suffer from chronic fatigue syndrome [14]. Patients were labeled pervasively passive, active, or moderately active based on their total amounts of physical activity. Such “sub-typing” can help to personalize interventions and define helpful and realistic treatment goals.

Other, more specific physical behavior measures than total amounts of physical activity are increasingly acknowledged as important and clinically relevant. Firstly, benefits of exercising or performing higher intensity activities are substantiated for cancer survivors [10]. Secondly, deteriorating effects of high amounts of sedentary behavior are increasingly acknowledged [15, 16] and addressed in many guidelines for cancer survivors [13]. Thirdly, measures that quantify distributions of these behaviors over time [17–19] were able to differentiate patients with chronic health conditions comparable to CCRF from healthy subjects independently of the total amounts of these physical behaviors. Consequently, focusing solely on total amounts of physical activity is probably too generic.

To acknowledge the relevance of different measures of physical behavior, Thompson et al. advocate to use profiles to describe physical activity of individuals [20]. This inherently leads to the research question in the current paper: What physical behavior profiles are prominent in persons who suffer from CCRF? This paper explicitly focusses on the interrelatedness of a range of physical behavior measures, which is novel in this field.

Secondly, this paper explores if the physical behavior profiles are related to participant characteristics. Therefore, demographic and clinical factors (age, sex, education, body mass index (BMI), working hours, cancer treatment types, time since last cancer treatment, and limitations due to pain or comorbidities), as well as fatigue, distress, and perceived work ability are studied for their relation with the physical behavior profiles.

## Methods

### Design

This study is a cross-sectional analysis of the baseline data of a randomized controlled trial to study Internet interventions for CCRF (approved by the Twente Medical Ethical Committee, Enschede, the Netherlands under number P12–26 and registered at The Netherlands National Trial Register (NTR3483, http://www.webcitation.org/6NWZqon3o)). Baseline data was assessed online on two occasions: during the registration process (T0a) and after eligibility was confirmed (T0b). At T0b, participants were asked to wear a hip-worn accelerometer for seven consecutive days, during waking hours, starting on a Friday and to keep activity diaries for the periods that they did not wear the accelerometer. Extensive information about the trial design is provided elsewhere [21].

### Participants

Of 179 patients who started the FNK-trial, seven did not provide sufficient accelerometer data, leaving 172 participants for the analyses (see Supplementary Materials, F1, for a flow chart). Participants were mostly women (72%), on average 56 years of age (between 21 and 82). Fifty-three percent of the participants acquired a college degree or higher. Most participants had breast cancer (46%) or hematological malignancies (17%). 9.4% had experienced recurrence of cancer, and 14% had metastasized cancer. Eight had reported cancer recurrence at the moment of submitting this paper.

### Measures

#### Physical Behavior Measures

##### Accelerometer Data Preprocessing

We focused on three dimensions of physical behavior: overall levels of physical activity, sedentary behavior (SB), and moderate-to-vigorous intensity physical activity (MVPA). These dimensions were operationalized as total amount measures, bout duration measures, and day part distribution measures. An overview is presented in the Supplementary Materials (T1).

The accelerometer (ProMove 3D, Inertia Technology, Enschede, the Netherlands, well described elsewhere [20]) outputs “integral of the modulus of acceleration” per minute in metric units (10^−3^ m/s^2^), which in this paper is referred to as counts per minute (cpm). The accelerometer data were scanned and processed in Matlab version R2013b (The MathWorks Inc., Boston, MA, USA). Non-wear was removed only when agreement was reached between two researchers (no agreement was reached in <1% of all measurement days). Reasons for missing accelerometer data were diverse (forgetting to charge or wear the system, esthetic objections, performing non-accelerometer-compatible activities such as swimming or sleeping, and various technical failures) but, unfortunately, sparsely provided in the activity diaries.

A measurement day was considered valid if it is consisted of >600 min of data. Three-day parts were distinguished: morning (05 h00 to 12 h00), afternoon (12 h00 to 18 h00), and evening (18 h00 to 00 h00). Day parts were considered valid if they are consisted of >120 min of data. An average of at least four valid days or day part combinations was required for analysis.

##### Amount Measures

Physical activity level (PAL (cpm)) was calculated by averaging all cpm values per day [22]. SB time (%) was the percentage of the total measurement time spent below 1303 cpm. Ninety-five percent of the distribution of SB in a lab study was captured below this value [23]. One thousand three hundred and three counts per minute is well below walking at a comfortable speed (*z* = −1.97) [20] and performing active office tasks (*z* = −2.58) [23]. MVPA time (%) was the percentage of the total measurement time spent above 2588 cpm. Two thousand five hundred eighty-eight counts per minute is the right limit of the 95% confidence interval of treadmill walking at 6 km per hour (2418 ± 275 cpm, *n* = 10) [24]. Two thousand five hundred eighty-eight counts per minute corresponds with a *z*-score of 0.36 of walking at a comfortable speed and a *z*-score of −0.32 of active office tasks.

##### Bout Duration Measures

Time of prolonged SB bouts (min) was the total SB time that was accumulated in bouts of 30 min and longer [25]. Time of prolonged MVPA bouts (min) was the total MVPA time that was accumulated in bouts of 10 min and longer [25].

##### Day Part Distribution Measures

For the day part difference of PAL (dPAL), the change score of the average PALs of two consecutive day parts was divided by the average PAL of the afternoon to correct for absolute difference of daily PALs between participants:

dPAL1 = ($$ \overset{-}{\mathrm{PAL}} $$_afternoon_ − $$ \overset{-}{\mathrm{PAL}} $$_morning_)/$$ \overset{-}{\mathrm{PAL}} $$_afternoon_

dPAL2 = ($$ \overset{-}{\mathrm{PAL}} $$_evening_ – $$ \overset{-}{\mathrm{PAL}} $$_afternoon_)/ $$ \overset{-}{\mathrm{PAL}} $$_afternoon_

For the day part difference of SB time (dSB), change scores between two consecutive day parts were calculated from the fractions (F) of the time of each day part that was spent sedentary:

dSB1 = F_afternoon_ – F_morning_

dSB2 = F_evening_ – F_afternoon_

#### Covariates

Fifteen factors were studied as covariates of the latent profiles, among which age**,** sex**,** BMI (calculated from self-reported height and weight), education (seven answers possible, dichotomized as accomplished college degree or higher), weeks since last primary cancer treatment (log transformed to account for skewness), and work status (dichotomized as working more than 8 h per week). Cancer treatments were categorized as chemotherapy, radiotherapy, and/or stem cell transplant. Comorbid conditions were categorized as (1) lung, (2) cardiovascular, (3) musculoskeletal, (4) neurologic, and (5) organ disease, (6) back and neck pain and trauma(tic) injuries, and (7) “other comorbidities” (mainly sleep apnea), and counted. Limitations by comorbid conditions were assessed: “How limiting are these complaints or disease(s) currently for you?” Answers ranged from *not at all* (1) to *very limiting* (4) and were dichotomized by a cutoff score ≥ 3. Limitations by pain were assessed: “In the past week, to what extend did you feel limited in performing daily physical activities because of pain?” Answers ranged from *not at all limited* (1) to *extremely limited* (7) and were dichotomized by a cutoff score ≥ 4.

Fatigue was assessed at T0b (Cronbach’s *α* = 0.839, *N* = 170) with the subscale fatigue severity of the Checklist Individual Strength [26, 27]. The sum score has been used in cancer survivors [1, 28] and has shown good internal consistency and discriminative validity [29]. Distress was assessed at T0a (Cronbach’s *α* = 0.883, *N* = 172) with the Hospital Anxiety and Depression Scale. The sum score has been validated thoroughly [30] and has been used in cancer survivor populations [31–34]. Perceived work-ability was assessed at T0b with the work ability index [35]: one question “Imagine that your working ability in the best period of your life is rated 10 points. How would you rate your working ability at the present moment?” that is answered on a scale from 0 (“not being able to work at all”) to 10.

##### Missing Covariate Data

The variables BMI and weeks since last treatment had missing values: 18 and 1 observations were missing, respectively. Furthermore, one participant did not finish the T0b assessment. Between samples *t* tests and chi-square tests revealed that participants with missing BMI’s reported lower perceived work ability (95% confidence interval of the difference (−1.694–0.001)), but were comparable on the other predictor variables.

### Statistical Analyses

The primary research question was answered with latent profile analysis with robust—full information—maximum log likelihood estimation in Mplus version 7.4 (Muthén and Muthén, Los Angeles, CA, USA). Indicators were *z*-scores of the physical behavior measures.

#### Model Checking

Three model series (models A_k_, B_k_, C_k_) were compared to decide how to deal best with theoretical pre-established overlap among the indicators (PAL with SB, pSB, MVPA, and pMVPA; MVPA with pMVPA; SB with pSB; dPAL1 with dSB1; and dPAL2 with dSB2), while acknowledging the distribution of pMVPA (left-skewed, 35 zero-observations). In each series, an ascending number of profiles was imposed (*K* = 2–5). In models A_k_, pMVPA was modeled as a left-censored variable, thus covariances with pMVPA were ignored. In models B_k_, pMVPA was log transformed. In models C_k_, overlap between the indicator variables was captured in four latent factors. The Supplementary Materials provide diagrams (F2) and Mplus syntax (T2) for all three model series.

### Latent Profiles Analysis

The results of the final model series were reported with Bayesian information criteria (BIC), class proportions, entropy, bootstrapped likelihood ratio tests, and Lo-Mendel-Rubin likelihood ratio tests. The best model from this series was established by evaluating the BIC [36] and reported by the profile means, standard deviations, and posterior probabilities for each profile.

### Covariates

The relation between the physical behavior profiles and participant characteristics was explored by means of Vermunt’s three-step approach [37], currently only available with list-wise deletion. The factor “stem cell transplant” had only one observation in one profile, and therefore was omitted. A backward elimination strategy was used. In each step, the factor with the highest *p* value of all lowest *p* values per factor was removed until only factors with a lowest *p* value below 0.01 remained. Omitting BMI resulted in the same final model.

## Results

### Model Checking

Not all models converged convincingly. Models A_4_ and A_5_ had no stable solutions, even when running 200.000 starts, and resulted in multiple errors and small profiles. Only one model (B_2_) from the B series and none from the C series resulted in stable solutions. The log transformation was not sufficient to specify pMVPA correctly in models B_k_, and models C_k_ were too complex for the amount of data. Therefore, we proceeded with the model A_k_ series, which converged adequately up to three classes.

### Latent Profiles Analysis

The results for the model A_k_ series are presented in Table 1. Model A_3_ was selected because that model had a lower BIC than the two-profile model. The A_3_ model resulted in profiles of 28, 71, and 73 participants in each profile, and most participants were allocated convincingly; 88% of the participants were allocated to a profile with probability higher than 0.8.

**Table 1:** Model results of the A_k_ series

*K*	BIC	Entropy	LMR (*p* value)	BLRT (*p*-value)	Profile proportions (*N*)
1	3435.299	Not applicable	Not applicable	Not applicable	172
2	3269.204	0.804	261.23 (.02)	<.001	72; 100
3	3218.251	0.848	147.25 (.23)	<.001	28; 71; 73
4^a^	Not available	0.869	Not performed	Not performed	7; 49; 55; 61
5^a^	Not available	0.901	Not performed	Not performed	4; 19; 40; 46; 63

*K* number of imposed profiles, *BIC* Bayesian information criterion, *LMR* Lo-Mendel-Rubin likelihood ratio test, *BLRT* bootstrapped likelihood ratio test

^a^No stable models were estimated; the models with best log likelihoods after 200,000 random starts are reported

**Fig. 1:**
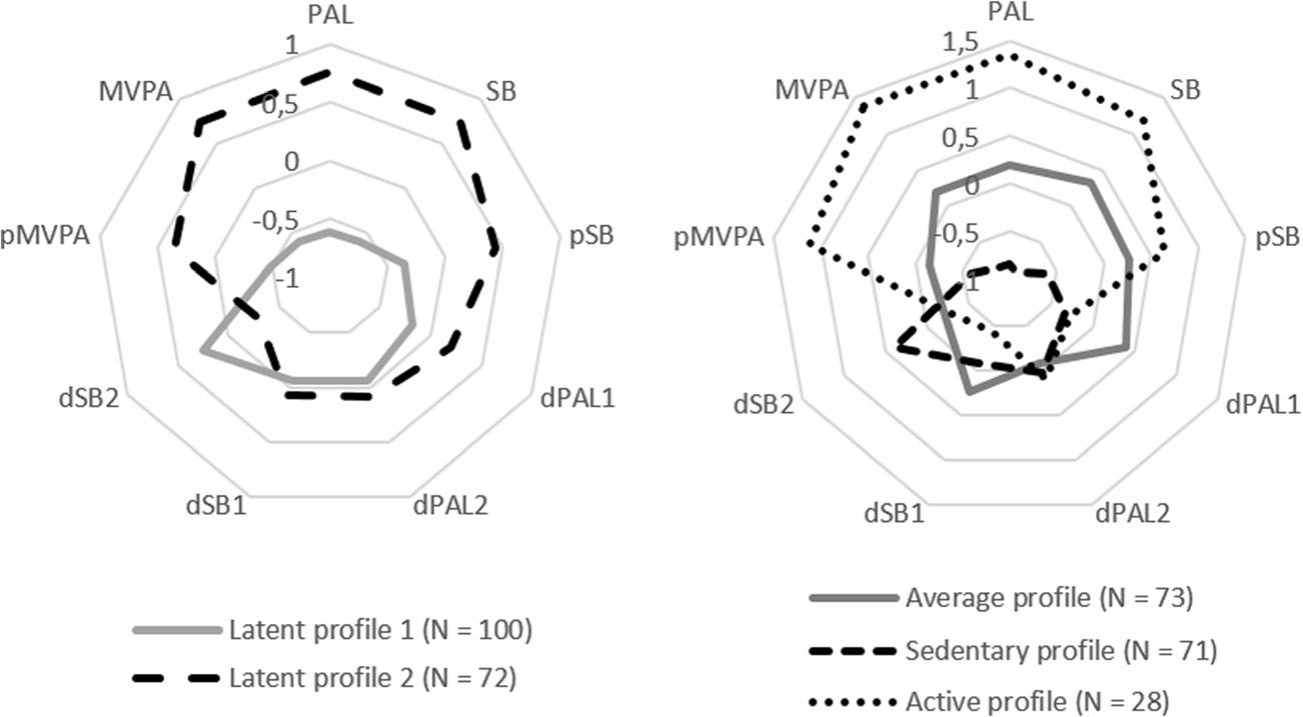
Standardized profile means (z-scores) of models A_2_ (*left*) and A_3_ (*right*). SB, dSB1, and dSB2 have switched signs, thus are defined “higher is better.” *PAL* physical activity level, *MVPA* moderate-to-vigorous intensity physical activity time, *pMVPA* prolonged bouts of MVPA, *SB* sedentary behavior time, *pSB* prolonged bouts of SB, *dPAL* and *dSB* (day part difference: *1* morning to afternoon, *2* afternoon to evening)

The profile means for model A_3_ (and for completeness also A_2_), standardized to *z*-scores of the total sample, are shown in Fig. 1. Raw profile means, standard deviations, and posterior probabilities are presented in Table 2. To improve readability, the A_3_ profiles are labeled “active”, “average”, and “sedentary”.

**Table 2:** Model results of the A_3_ model

	Total sample^c^		Sedentary		Average		Active	
*N* (%)			71 (41%)		73 (42%)		28 (16%)	
Profile posterior probabilities^b^, mean (lowest)	0.936		0.926 (.50)		0.944 (.59)		0.943 (.66)	
Profile posterior probabilities <0.8, *N* (%)	20 (12%)		11 (15%)		7 (10%)		2 (7%)	
	mean	stdev	mean	stdev	mean	stdev	mean	stdev
Physical activity level (cpm) (*N* = 165)	790.3	217.0	609.7	71.0	834.1	43.6	1081.5	103.7
MVPA (%) (*N* = 165)	6.22	3.47	0.10	0.62	13.06	1.38	27.94	2.43
Prolonged bouts of MVPA (min) (*N* = 165)^a^	10.64 ^a^	12.62	3.14	5.36	8.87	8.77	24.82	28.35
Sedentary behavior time (%) (*N* = 165)	78.36	7.21	84.84	2.09	76.02	2.39	69.85	4.18
Prolonged bouts of SB (min) (*N* = 165)	325.6	110.6	391.3	88.7	295.8	84.7	254.5	56.8
dPAL1 (%-pt) (*N* = 148)	−17.47	35.07	−28.83	41.70	−3.72	17.7	−27.15	30.5
dPAL2 (%-pt) (*N* = 166)	−28.80	22.49	−28.21	26.36	30.30	20.0	−26.32	21.60
dSB1 (%-pt) (*N* = 148)	2.1	8.4	2.8	5.0	0.1	9.4	5.7	10.1
dSB2 (%-pt) (*N* = 166)	10.2	7.1	7.5	4.38	11.8	6.7	12.3	8.3

*stdev* standard deviation, *MVPA* moderate-to-vigorous intensity physical activity time, *dPAL* and *dSB* day part difference (1: morning to afternoon, 2: afternoon to evening)

^a^Skewness = 2.13; kurtosis = 6.66; median = 6.57. 15% of the total sample (*n* = 25) accrued >21 min of pMVPA per day, thus potentially accrues 150 min of pMVPA per week.

^b^The profile mean (of those participants who were actually assigned to this specific profile) of the posterior probabilities for each profile. Between brackets is the lowest posterior probability with which a participant was assigned to that profile

^c^ Supplementary Materials (T4) shows the bivariate covariances matrix and modeled covariances on the overall level of models A_1_ and A_3_

The right panel of Fig. 1 shows that the mean scores of the average profile are similar to the sample mean scores (*z* < |0.5|) for all indicators. The sedentary and active profile can be distinguished best from the average profile by PAL and MVPA. However, differences between profiles are also captured in the mean scores of other measures. For example, decline of physical activity from morning to afternoon was lowest (so high dPAL1) in the average profile. By contrast, dPAL2 was not distinctive at all. The Supplementary Materials (F3) show histograms of the sample profile distributions, which provide further insight into the distinctive character of all indicator measures.

### Covariates

Exploration of the relation between the physical behavior profiles and participant characteristics showed that participants in the sedentary profile were older and less likely to report limitations by comorbid conditions compared to the average profile and were more likely to have limitations by pain compared to participants in the active profile. The results are presented in Table 3.

**Table 3:** Predictive value of participant characteristics of the three-profile model

	Total sample	Eliminated in step (lowest *p* value)	Average compared to sedentary^a^	Active compared to sedentary^a^	Active compared to average^a^
Age (years)	55.8 (10.2)		**−0.070 (.001)**	−.047 (.105)	−0.024 (.372)
Sex (male)	28%	2 (.698)			
Education (≥ college degree)	52.9%	3 (.581)			
Work status (> 8 h/week)	53.8%	6 (.423)			
Body mass index (kg/m^2^) (*N* = 154)	26.4 (5.1)	9 (.061)			
Weeks since last treatment^b^	206 (236)	7 (.248)			
Comorbid conditions (≥ 2)	14.0%	4 (.465)			
Limitations by comorbid condition (≥ 3/4)	37.8%		**1.496 (.002)**	1.48 (.011)	0.015 (.977)
Limitations by pain (≥ 4/7)	32.8%		−0.923 (.046)	**−1.959 (.006)**	1.035 (.136)
Treatment: chemo	69.6%	8 (.27)			
Treatment: radiotherapy	59.7%	1 (.717)			
Treatment: stem cell transplant	6.4%	0^c^			
Fatigue (8–56)	42.0 (8.0)	11 (.010)			
Distress (0–42)	14.3 (6.8)	5 (.474)			
Work ability (0–10)	3.2 (1.7)	10 (.035)			

^a^Values are reported as logodds (*p* value). Logodds > 0 indicate that the risk of the outcome falling in the comparison profile relative to the risk of the outcome falling in the referent profile increases as the variable increases. Univariate results are presented in the Supplementary Materials (T*5*)

^b^Median: 126 weeks

^c^In the active profile, only one participant had experienced a stem cell transplant; therefore, this factor was excluded from the analyses

## Discussion

In this study, multiple physical behavior measures were collected to exhibit heterogeneity of patients who suffer from CCRF by means of physical behavior profiles. Three profiles were distinguished: a sedentary, active, and average profile. Furthermore, we investigated participant characteristics as covariates of these profiles, to increase knowledge on and enhance personalization of interventions that somehow target physical behavior in this population.

The three profiles were mostly distinguished by the measures PAL and MVPA time. Compared to the sedentary profile, participants in the active profile were roughly twice as active in terms of PAL and spent almost seven times longer on prolonged MVPA. Time spent in prolonged bouts of SB also differed between profiles: means were 4 h and 15 min in the active profile, compared to 6 h and 31 min in the sedentary profile. The average profile had the lowest (almost no) decline of PAL between morning and afternoon. These results show that persons who suffer from CCRF form a very heterogeneous group in terms of physical behaviors, who require diverse intervention goals when focusing on physical behavior.

All three profiles provide potential focus for intervening on physical behavior in clinical practice. Obvious goals—and currently widely accepted [13]—are increasing PAL and prolonged MVPA time, which apply to participants in the sedentary and average profile. However, in the active profile, increasing PAL is expected to be less effective compared to the other profiles. Patients who have an active profile might benefit from reducing the time of pronged SB or from better dividing physical activities throughout the day by energy conservation strategies [38]. Indeed, a patient in the active profile may actually be helped by reducing PAL before increasing it gradually in order to match the patient’s physical behavior to his or her capacity. This assumption is supported by a study in breast cancer survivors in which self-reported physical activity measures showed that psychological outcomes were poor for the quartile of patients with the highest durations of physical activity [39].

Boundaries between the physical behavior profiles are not strict, which hampers direct transfer to clinical practice; 88% of the participants were assigned to a profile with a probability above 80%. The distinct profiles can provide a direction for defining an effective physical behavior goal, but encourage to match the intervention goals to the patient’s perspective and individual wishes as well.

The second research question focused on covariates of these physical behavior profiles. Participants who reported stronger limitations by pain were more likely to have a sedentary profile compared to an active profile. This result highlights the relevance of pain management in the context of physical activity interventions as it could be a barrier for becoming more physically active and for staying physically active when professional guidance in the context of rehabilitation stops.

Additionally, older participants and participants who reported no or only weak limitations due to comorbid conditions were more likely to have a sedentary profile compared to an average profile. Studies on—not necessarily fatigued—breast cancer survivors support the results on the associations with age [40]. The result on the association with limitations by comorbid conditions seems contradictory to the findings on limitations by pain. An explanation might be that comorbid conditions are perceived as less limiting for those who are engaged in a sedentary lifestyle, although it should be noted that the question that assesses limitations due to comorbid conditions do not explicitly mention physical activities.

Our sample is comparable to a sample of long-term colon cancer survivors [25], in terms of prolonged MVPA. Fourteen percent compared to 15% in the current sample meets clinical guidelines, operationalized as spending 150 min per week on MVPA in bouts of 10 min or longer. However, MVPA time (6.2%) differed greatly from samples of breast cancer survivors: 1.9% [16] and 1.1% [15]. Also, SB time (78.4% in our sample) differed from breast cancer survivors (61.3% [41], 66.3% [15], and 78.2% [16]) and from colon cancer survivors (60.7% [25]). Prolonged SB differed even more: 152.9 min of prolonged SB [25] versus 325.6 min in the current sample. Differences could relate to choices for cutoff values, as well as to clinical status; the comparison samples were not necessarily fatigued and homogeneous groups in terms of cancer diagnoses.

Our study has a number of limitations. Firstly, all participants were willing to follow an intervention in a trial called “Fitter after cancer” and were aware that physical activity was measured. Both properties could provide bias by overestimating PAL compared to the general population of CCRF. Secondly, generalizability might be hampered because our sample was diagnosed with various cancer types and received different treatments. Thirdly, evaluating 1-minute measurement intervals, although generally used [42], causes real life data points to represent a mixture of behaviors. Therefore, absolute values of SB and MVPA measures should be interpreted cautiously. Finally, in order to come to the set of physical behavior measures, some measures and cutoff values for time or cpm lack evidence or were chosen arbitrarily. However, by describing these choices transparently, we presume that the results of this study are valuable nevertheless.

## Conclusions

Three profiles of physical behavior were distinguished in a sample of severely fatigued cancer survivors, showing the heterogeneous character of the sample. The results indicate that optimal support might require substantially different treatment goals for different patients. These profiles demonstrate an opportunity for personalizing physical behavior oriented treatment goals, but further research should test the applicability of these profiles in clinical practice.

## Electronic supplementary material

ESM 1 (PDF 636 kb)
