# Supplementary material for: Physical Behavior Profiles in Chronic Cancer-Related Fatigue
Source: Int J Behav Med. 2017 Jul 11;25(1):30–7. doi: 10.1007/s12529-017-9670-3 (PMC5803288; doi:10.1007/s12529-017-9670-3)
Supplement: Supplementary file 1 — (PDF 636 kb) [file 12529_2017_9670_MOESM1_ESM.pdf]

Supplementary materials

MDJ Wolvers, JBJ Bussmann, FZ Bruggeman-Everts, ST Boerema, R van de Schoot, MMR Vollenbroek-Hutten. Physical behavior profiles in chronic cancer-related fatigue. International Journal of Behavioral Medicine. 2017

Corresponding author: MDJ Wolvers; m.wolvers@rrd.nl

Supplementary Table 1. Operationalization of physical behavior measures.

	Amount	Bout duration	Day part distributions[a]
Overall physical activity level	Physical activity level, average count/minute (PAL [cpm])[b]. Also considered total counts/day (r=.93)[c]	Not applicable.	dPAL1 and dPAL2. Corrected for total PAL.
Sedentary behavior	Sedentary behavior time/measured minutes (SB [%])[b]. Also considered total sedentary time/day (r=.73)[c].	Sedentary time accumulated in prolonged bouts (>30 minutes) (pSB [min])[d]. Also considered W_{50} (r=.93)[c].	dSB1 and dSB2. Corrected for total sedentary behavior time.
Moderate-to-vigorous intensity physical activity (MVPA)	MVPA intensity time/measured minutes (MVPA [%])[b]. Also considered total MVPA time/day (r=.98)[c].	MVPA time accumulated in prolonged bouts (> 10 minutes) (pMVPA [min])[d]. Also considered W_{50} (r=.78)[c].	Not applicable[e].

Measures that combine bout frequency and bout duration (hybrid measures) and bout duration distribution measures (Chastin 2010) were not considered as indicators because they can be difficult to interpret.
Abbreviations: dPAL and dSB: day parts difference of PAL and SB respectively (1: morning to afternoon; 2: afternoon to evening); cpm: counts per minute.
[a]Three day parts were defined: morning (05h00 and 12h00), afternoon (12h00 and 18h00), evening (18h00 and 00h00).
[b]Accounting for variation in awake and/or measured minutes.
[c]Bivariate correlations (r) were calculated between the potential measures within a cell. All correlations were significant (*p*<.001).
[d]Ease of interpretation was preferred.
[e]Not enough observations of MVPA-minutes in day-parts.

Supplementary Table 2. Mplus syntax of models A, B, and C.

Series A	Series B	Series C
Data:<br>File = "BaselinePAdata.dat";<br><br>Variable:<br>names = PAL PI MVPA sMVPA<br>sPI DP1 DP2 PIR1 PIR2;<br>usevariables = PAL - PIR2;<br>missing = all (-999);<br>classes = c(3); !for k = 1 to 5<br>censored = sMVPA (b);<br><br>Define:<br>!for interpretation purposes.<br>dp1 = -dp1;<br>dp2 = -dp2;<br>pi = -pi;<br>spi = -spi;<br><br>Analysis:<br>type = mixture;<br>estimator = MLR;<br>starts = 200 40;<br><br>Model:<br>%overall%<br>PI with sPI;<br>PAL with sPI PI MVPA;<br>dp1 with pir1;<br>dp2 with pir2;<br><br>%c#1%<br>PAL - PIR2;<br>%c#2%<br>PAL - PIR2;<br>%c#3%<br>PAL - PIR2;	Data:<br>File = "BaselinePAdata.dat";<br><br>Variable:<br>names = PAL PI MVPA sMVPA<br>sPI DP1 DP2 PIR1 PIR2;<br>usevariables = PAL to PIR2;<br>missing = all (-999);<br>classes = c(3); !for k = 1 to 5<br><br>Define:<br>!for interpretation purposes.<br>dp1 = -dp1;<br>dp2 = -dp2;<br>pi = -pi;<br>spi = -spi;<br><br>Analysis:<br>type = mixture;<br>estimator = MLR;<br>starts = 200 40;<br><br>Model:<br>%overall%<br>PI with sPI;<br>PAL with sPI PI MVPA;<br>PAL with sMVPA;<br>MVPA with sMVPA;<br>dp1 with pir1;<br>dp2 with pir2;<br><br>%c#1%<br>PAL - PIR2;<br>%c#2%<br>PAL - PIR2;<br>%c#3%<br>PAL - PIR2;	Data:<br>File = "BaselinePAdata.dat";<br><br>Variable:<br>names = PAL PI MVPA sMVPA<br>sPI DP1 DP2 PIR1 PIR2;<br>usevariables = PAL - PIR2;<br>missing = all (-999);<br>classes = c(3); !for k = 1 to 5<br>censored = sMVPA (b);<br><br>Define:<br>!for interpretation purposes.<br>dp1 = -dp1;<br>dp2 = -dp2;<br>pi = -pi;<br>spi = -spi;<br><br>Analysis:<br>type = mixture;<br>estimator = MLR;<br>starts = 200 40;<br>algorithm = integration;<br>integration=montecarlo;<br><br>Model:<br>%overall%<br>f1 by PAL MVPA smvpa;<br>f2 by DP1 pir1;<br>f3 by DP2 pir2;<br>f4 by PAL pi spi;<br><br>f1-f4@1;<br>[f1-f4@0];<br>[zpal-zpir2*];<br>f1-f4 with f1-f4@0;<br>f1-f4 with c@0;<br><br>%c#1%<br>PAL - PIR2;<br>%c#2%<br>PAL - PIR2;<br>%c#3%<br>PAL - PIR2;

Supplementary Table 3. Z-scores of A_3 model.

	Sedentary		Average		Active	
	mean	stdev	mean	stdev	mean	stdev
PAL [cpm] (N=165)	-0.832	0.327	0.202	0.201	1.342	0.478
MVPA [%] (N=165)	-0.835	0.178	0.192	0.396	1.371	0.701
pMVPA [min] (N=165)[a]	-0.594	0.425	-0.140	0.695	1.124	2.246
SB [%] (N=165)	0.835	0.290	-0.325	0.331	-1.180	0.580
pSB [min] (N=165)	0.898	0.802	-0.269	0.766	-0.642	0.513
dPAL1 [%-pt] (N=148)	-0.324	1.189	0.392	0.505	-0.276	0.870
dPAL2 [%-pt] (N=166)	0.026	1.172	-0.067	0.888	0.110	0.960
dSB1 [%-pt] (N=148)	0.079	0.592	-0.243	1.120	0.428	1.207
dSB2 [%-pt] (N=166)	-0.388	0.620	0.223	0.953	0.292	1.178

Abbreviations: stdev: standard deviation, PAL: physical activity level; MVPA: moderate-to-vigorous intensity physical activity time; pMVPA: prolonged bouts of MVPA; SB: sedentary behavior time, pSB: prolonged bouts of SB; dPAL and dSB: day part difference (1: morning to afternoon, 2: afternoon to evening).

[a]Skewness = 2.13; kurtosis = 6.66.

Supplementary Table 4. Modeled and bivariate correlations of the indicators.

	sMVPA	MVPA	PAL	SB	pSB	dPAL1	dPAL2	dSB1	dSB2
sMVPA	1								
MVPA	0.534	1	0.346 (A_1)<br>0.094 (A_3)						
PAL	0.418	0.852	1	0.631 (A_1)<br>0.214 (A_3)	0.528 (A_1)<br>0.259 (A_3)				
SB	-0.26	-0.806	-0.916	1	0.786 (A_1)<br>0.379 (A_3)				
pSB	-0.07	-0.539	-0.72	0.792	1				
dPAL1	0.052	0.093	0.172	-0.128	-0.172	1		0.804 (A_1)<br>0.702 (A_3)	
dPAL2	0.076	0.142	0.131	-0.065	-0.066	-0.245	1		0.764 (A_1)<br>0.746 (A_3)
dSB1	-0.048	0.031	0.016	-0.098	-0.07	-0.809	0.206	1	
dSB2	0.043	0.208	0.225	-0.315	-0.205	0.378	-0.771	-0.382	1

The upper triangle presents the overall-level correlation of the A_1 and A_3 models, the lower triangle presents bivariate correlations.

Abbreviations: PAL: physical activity level; MVPA: moderate-to-vigorous intensity physical activity time; pMVPA: prolonged bouts of MVPA; SB: sedentary behavior time, pSB: prolonged bouts of SB; dPAL and dSB: day part difference (1: morning to afternoon, 2: afternoon to evening).

Supplementary Table 5. Predictive value of participant characteristics of the three-profile model.

	Univariate				Multivariate		
	Average compared to sedentary[a]	Active compared to sedentary[a]	Active compared to average[a]	Eliminated[b] (lowest *p*-value)	Average compared to sedentary[a]	Active compared to sedentary[a]	Active compared to average[a]
Demographic and clinical factors							
Age [years]	-0.067 (.001)	-0.041 (.129)	0.026 (.325)	In final model	-0.070 (.001)*	-0.047 (.105)	-0.024 (.372)
Sex (male)	-0.05 (.905)	0.201 (.697)	0.251 (.638)	2 (.698)			
Education (≥ college degree)	-0.067 (.858)	0.002 (.997)	0.069 (.890)	3 (.581)			
Work status (> 8 h/week)	-0.562 (.150)	0.211 (.666)	-0.033 (.947)	6 (.423)			
Body mass index (N = 154)	-0.086 (.039)	-0.075 (.139)	0.011 (.845)	9 (.061)			
Weeks since last treatment[c]	-0.368 (.360)	-0.217 (.709)	0.150 (.808)	7 (.248)			
Comorbid conditions (≥ 2)	-0.746 (.160)	-1.242 (.151)	-0.496 (.595)	4 (.465)			
Limitations by comorbid condition	1.127 (.007)	0.876 (.089)	-0.251 (.613)	In final model	1.496 (.002)*	1.480 (.011)	0.015 (.977)
Limitations by pain (≥ 4/7)	-0.562 (.150)	-1.561 (.019)	-1.000 (.148)	In final model	-0.923 (.046)	-1.959 (.006)*	1.035 (.136)
Treatment: chemo	-0.400 (.331)	-0.156 (.773)	0.244 (.653)	8 (.27)			
Treatment: radiotherapy	-0.534 (.170)	-0.488 (.328)	0.047 (.926)	1 (.717)			
Treatment: stem cell transplant	-0.500 (.498)	-0.939 (.437)	-0.439 (.734)	0[d]			
Psychological factors							
Fatigue severity [8-56]	-0.056 (.018)	-0.045 (.11)	0.011 (.712)	11 (.010)			
Distress [0-42]	0.021 (.446)	0.022 (.553)	0.000 (.998)	5 (.474)			
Work ability [0-10]	0.054 (.655)	0.239 (.070)	0.184 (.122)	10 (.035)			

[a]Values are reported as logodds (*p*-value). Logodds > 0 indicate that the risk of the outcome falling in the comparison profile relative to the risk of the outcome falling in the referent profile increases as the variable increases.
[b]This column indicates in which step each variable was eliminated from the prediction model (the factor of which the lowest *p*-value is the highest of all other lowest *p*-values).
[c]Median time since treatment is 126 weeks.
[d]The active profile had only one participant who experienced a stem cell transplant, therefore this factor was excluded from the multivariate analyses.
*$p < .01$

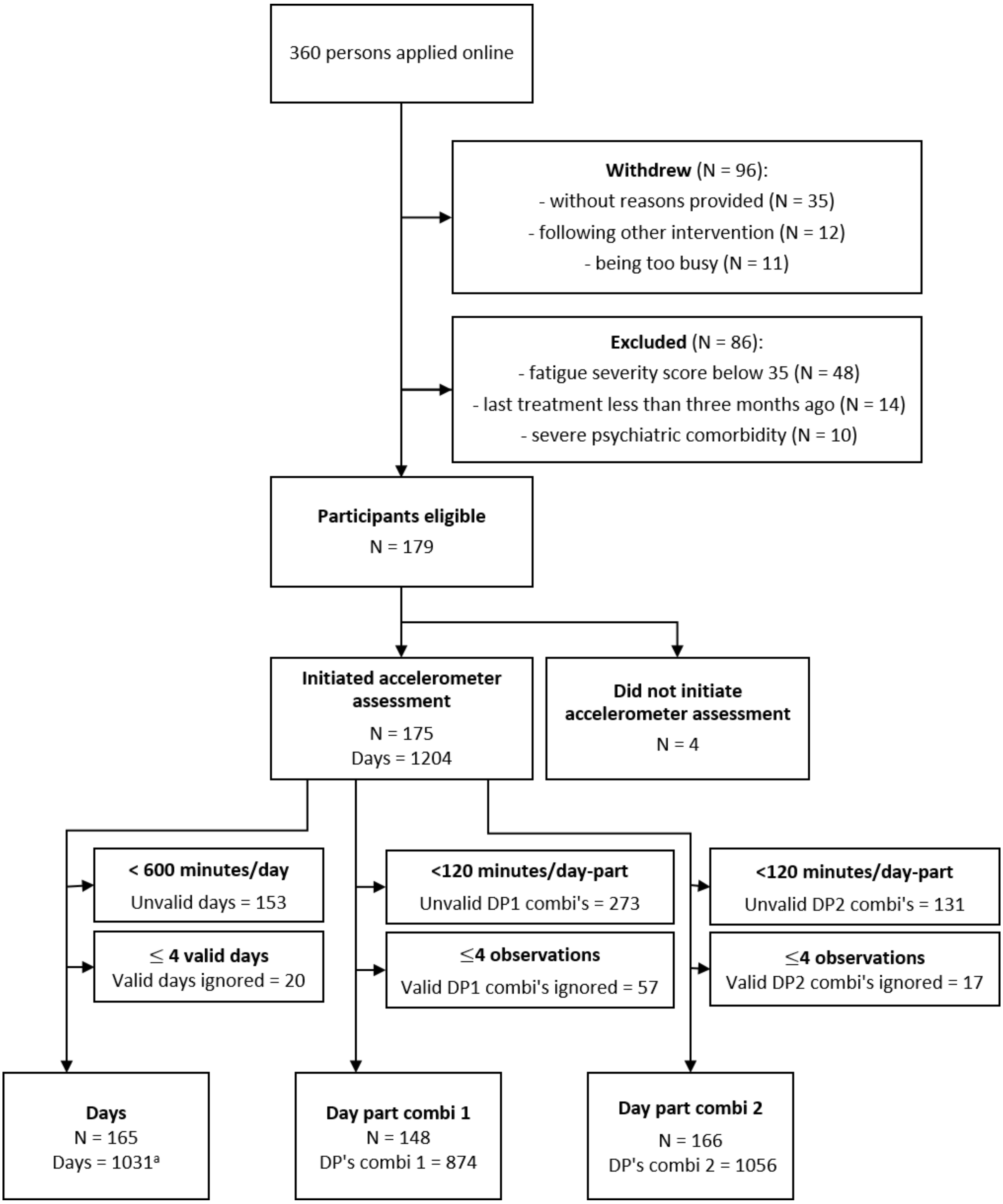

Supplementary Figure 1. Flow chart of selecting accelerometer data.

Data was selected in two steps. In the first step, valid days or day part combinations were selected, in the second step the number of observations per participant. 172 participants had sufficient data on at least one PB-measure. Abbreviations: DP combi: day part combination (1 = morning to afternoon, 2 = afternoon to evening).

[a]Wear time per valid day, specified by the number of valid days that a participant provided, was on average 612 (4 valid days, n = 8), 695 (5 valid days, n = 25), 737 (6 valid days, n = 50), and 814 (7 valid days, n = 82) minutes.

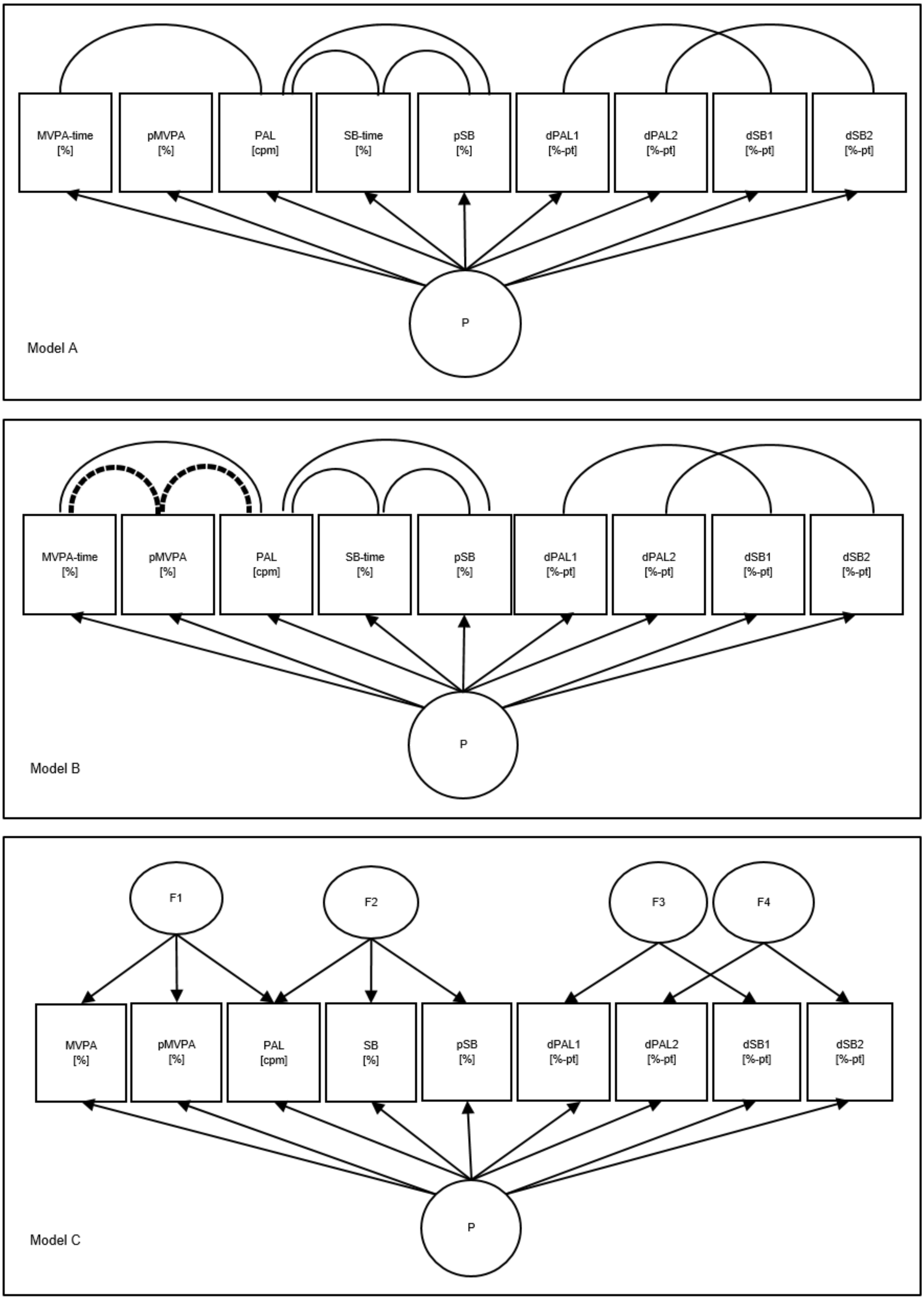

Supplementary Figure 2. Diagram of the latent profiles models A, B, and C.

P is a categorical latent variable (latent profiles factor) that ranges between 1 and 5, depending on the number of profiles that is imposed at the model. The indicators are physical behavior measures: moderate-to-vigorous physical activity time (MVPA-time), prolonged bouts of MVPA (pMVPA), physical activity level (PAL), sedentary behavior time (SB), prolonged bouts of SB (pSB), dPAL and dSB: day-parts distributions (1: morning to afternoon; 2: afternoon to evening). Curved, dashed lines are correlations between the residual variances of the indicators which were left out for simplicity.

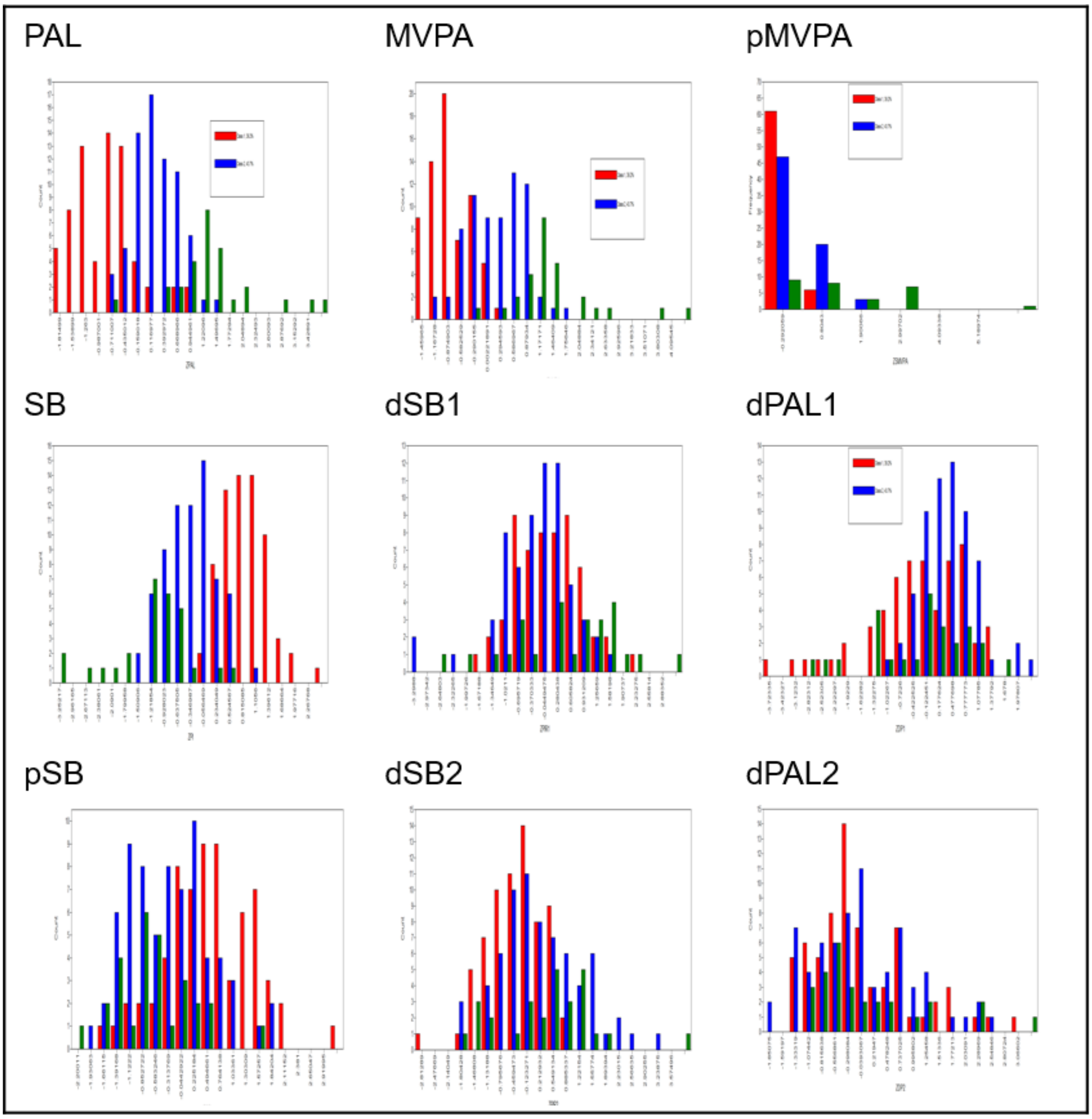

Supplementary Figure 3. Sample indicator distributions for the latent profiles of model A3.
The indicators are all physical behavior measures: moderate-to-vigorous physical activity time (MVPA-time), prolonged bouts of MVPA (pMVPA), physical activity level (PAL), sedentary behavior time (SB), prolonged bouts of SB (pSB), dPAL and dSB: day-parts distributions (1: morning to afternoon; 2: afternoon to evening).

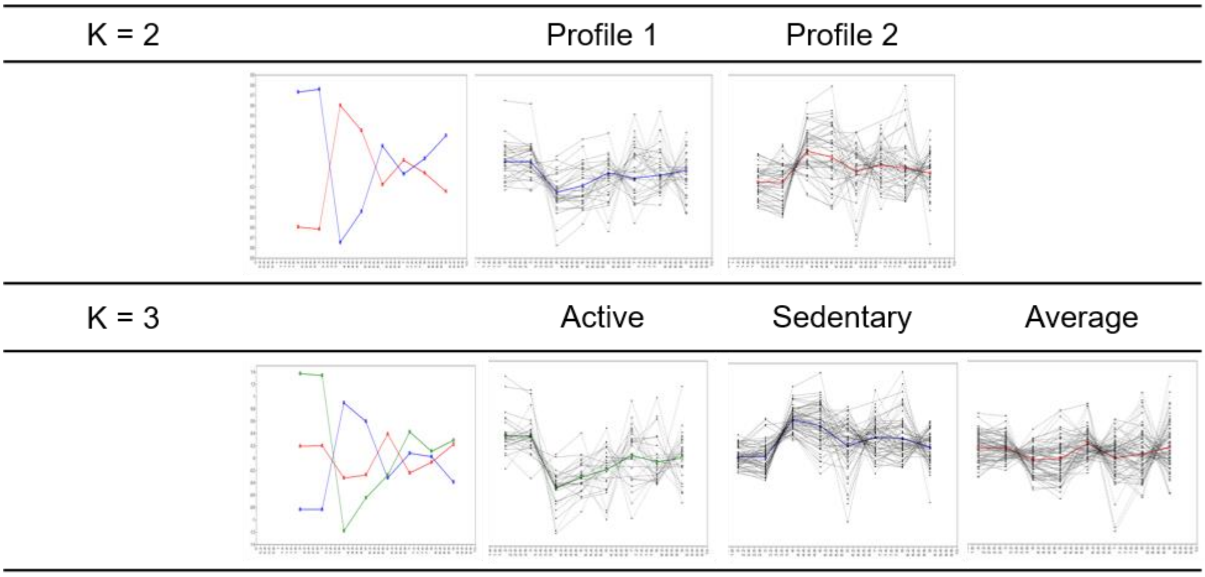

Supplementary Figure 4. Model results of the A series: standardized profile means and actual scores [Z-scores] of the indicators.

Left-to-right: MVPA (2) PAL (3) PI (4) pPI (5) dPAL1 (6) dSB1(7) dPAL2(8) dSB2(9). MVPA (2): moderate-to-vigorous-intensity physical activity; PAL (3): physical activity level; SB (4): sedentary behavior time; pSB (5): prolonged bouts of SB >30 min; dPAL1: percentual difference of PAL, morning to afternoon; dSB1: percentual difference of SB, morning to afternoon (7), dPAL2 (8): dPAL, afternoon to evening; dSB2 (9): dSB, afternoon to evening.
